# Cow's Milk Positively Impacts Bone Formation by Regulating the Osteocalcin Pathway Compared to Transgenic and Non‐Transgenic Soy‐Based Beverages in BALB/c Mice

**DOI:** 10.1002/mnfr.70369

**Published:** 2026-01-16

**Authors:** Eduarda Pires Costa, Mariáurea Matias Sarandy, Amanda Alves Lozi, Maria Tatiana Soares Martins, Patricia da Silva Mattosinhos, Manoela Maciel dos Santos Dias, Sergio Luis P. Matta, Romulo Dias Novaes, Reggiani Vilela Gonçalves

**Affiliations:** ^1^ Department of General Biology Federal University of Viçosa Viçosa Minas Gerais Brazil; ^2^ Department of Animal Science Plants For Human Health Institute North Carolina State University North Carolina Research Campus Kannapolis North Carolina USA; ^3^ Department of Animal Biology Federal University of Viçosa Viçosa Minas Gerais Brazil; ^4^ Institute of Biomedical Sciences Federal University of Alfenas Alfenas Minas Gerais Brazil

**Keywords:** bone, milk, nutrition, osteocalcin, soy

## Abstract

Bone health is influenced by nutrition, with milk being one of the main dietary sources of calcium. However, many people are reducing their milk intake in favor of plant‐based alternatives. This study compares the effects of cow's milk and soy‐based beverage supplementation on femur morphofunctional parameters in BALB/c mice. The animals were randomized into four groups treated for 42 days with distilled water (G1), non‐transgenic soy drink (G1), transgenic soy drink (G1), and cow's milk (G1). After euthanasia, femurs were collected for morphological and biomechanical analyses. G3 animals exhibited reduced phosphorus levels, cortical and trabecular thickness, diaphyseal diameter, type I collagen content, and increased porosity in the cortical bone. Conversely, G4 animals showed greater trabecular density (+20%), bone area (+20%), and trabecular width (+150%), as well as lower volume density of the medullary canal and trabecular separation. These animals also exhibited increased osteocalcin immunostaining, magnesium, phosphorus, and calcium levels; reduced diaphyseal porosity; and increased bone weight, suggesting that cow's milk positively impacts bone formation by stimulating osteocalcin production (+5%), especially compared to transgenic soy‐based beverages. Therefore, our findings indicate that cow's milk may offer a safer alternative to soy‐based beverages for promoting bone health.

## Graphical abstract

Comparison of the effects of cow's milk and transgenic soy‐based beverages on bone health in BALB/c mice. Cow's milk promoted higher trabecular density; improved bone structure; and increased osteocalcin, calcium, and phosphorus levels. The non‐transgenic soy beverage led to reduced cortical thickness and increased diaphysis diameter, while the transgenic soy beverage resulted in decreased diaphysis diameter, cortical bone thickness, type I collagen content, and increased pore formation.

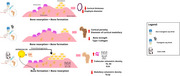

**Abbreviations**
B.ar: bone area
CT: cortical thickness
DB: bone diameter
DM: diameter of the medullary canal
Tb.Sp: trabecular separation
Tb.Witrabecular width: trabecular width
Vv bone/marrow: volumetric density of trabecular bone or bone marrow

## 1 Introduction

Changes in eating habits have raised concerns about bone health, as a balanced diet is essential for the development and maintenance of bone mass and its biomechanical function [1]. Accordingly, bone metabolism, microstructure, matrix mineralization, and bone mineral density are directly influenced by nutritional factors, especially dietary composition. As an important component included in both Eastern and Western diets, cow's milk stands out for its impact on bone health [2] as an important source of calcium, vitamin D, and proteins, which help regulate bone remodeling and reduce bone loss [3, 4]. Studies have shown that dairy consumption provides antioxidant, antimicrobial, and anticarcinogenic benefits, which are largely attributed to bioactive milk peptides and proteins [5, 6]. Reduced or absent dairy consumption has been linked to an increased risk of osteoporosis and osteopenia, affecting quality of life in all age groups [7]. The Osteoporosis Foundation estimates that osteoporosis causes more than 8.9 million fractures annually in Europe, with a global economic burden of 52 billion dollars in 2019 [8].

Milk consumption, however, has decreased due to increased lactose intolerance diagnoses, adherence to vegan diets, allergies, and calorie concerns [9, 10]. Plant‐based beverages, particularly soy‐based alternatives, have gained popularity as lactose‐free and cholesterol‐free substitutes for cow's milk [11]. Soy provides a high‐quality amino acid profile [12] and is rich in flavonoids, known for their therapeutic potential in inflammatory and oxidative stress‐related conditions [13]. Studies indicate that soy proteins and isoflavones can improve trabecular bone parameters, including trabecular number, bone volume, and mineral density [14, 15]. Still, the nutritional composition of these beverages varies depending on the raw materials, processing, storage, and fortification [16]. The increasing demand for soybeans has led to the development of genetically modified (GM) variants to enhance yield and reduce costs [17]. These modifications confer herbicide resistance, preventing crop losses due to competing weeds. However, genetic modification may also lead to unintended changes in the nutritional profile and bioactive compounds, such as proteins and isoflavones, which could influence biological responses to soy consumption. Although the health effects of GM soybeans remain uncertain, some studies suggest potential risks, including hepatic, pancreatic, and gonadal toxicity in animal models [18, 19]. In contrast, organic soybeans are cultivated using sustainable practices without chemical inputs, preserving their composition but resulting in lower productivity and higher costs [20].

Although some studies have examined the effects of cow's milk and soy beverages on human health, the findings remain inconclusive and do not provide definitive answers, especially regarding the key pathways activated after exposure. Furthermore, no studies have explored the effects of different soy beverages (e.g., based on transgenic and non‐transgenic varieties) compared to cow's milk on bone health. Therefore, this study is the first to combine biometric, biomechanical, histomorphometric, and immunofluorescence analyses to assess bone health. In addition, bone matrix composition and the ultrastructural aspects of the femurs in BALB/c mice were studied. The objective was to understand the main activated cellular pathways potentially related to bone microstructural and biomechanical adaptations in response to milk intake. These findings provide relevant insights into the comparative effects of cow's milk and transgenic soy beverages on bone health, which may inform future dietary recommendations.

## 2 Materials and Methods

### 2.1 Animals

Fifty‐day‐old male BALB/c mice (about 30 g), were obtained from the Central Animal House of the Biological and Health Sciences Center of the Federal University of Viçosa. The animals received standard rodent food and water ad libitum and were supplemented with non‐transgenic soy drink, transgenic soy drink, or cow milk once a day. The animals were housed in individual cages maintained in experimental facilities with photoperiod (12/12‐h light/dark cycles) and temperature (21 ± 1°C) controlled. Body mass was recorded weekly. This study was approved by the Animal Ethics Committee of the Federal University of Viçosa (registration number 23/2022).

### 2.2 Experimental Model and Diets

Twenty‐eight mice were randomized into four groups with seven animals per group. The groups (G) received drinking water (G1), non‐transgenic soy drink (G2), transgenic soy drink (G3), or cow milk (G4) once a day. All treatments were administered by gavage for 42 days. Although 42 days were sufficient to observe preliminary effects, this period is relatively short for full bone remodeling, which may limit the detection of long‐term structural and biomechanical changes. Milk (0.7 mL) was administered once daily at 24.03 mg/kg body weight in mice, which corresponds to the equivalent human dose of 1.95 mg/kg. The human‐to‐mouse dose conversion was calculated using body surface area normalization (Km factor: 3 for mouse, 37 for human) according to the method described by Reagan‐Shaw et al. [21]. The nutritional composition of the commercially available beverages used is detailed in Table 1. Although the nutritional composition of the beverages is provided in Table 1, a detailed biochemical characterization such as quantification of isoflavones, protein fractions, antinutritional compounds, or GM‐specific markers was not performed in this study. Future investigations should include these analyses to better understand the potential biological effects of transgenic and non‐transgenic soy beverages. Both transgenic and non‐transgenic soy‐based beverages (G2 and G3) were enriched with calcium, according to product labels. Cow's milk (G4) contains calcium naturally and serves as a reference for comparison. The animals were weighed before and after the experimental period. Twenty‐four hours after the last meal, the animals were anesthetized with tribromoethanol (250 mg/kg, i.p.) and euthanized by exsanguination via cardiac puncture. The femurs were collected for analysis.

**TABLE 1:** Nutritional information (whole cow's milk (1), transgenic soy drink (2), and non‐transgenic soy drink (3)). Values are expressed per 200 mL serving.

	Milk type					
Parameters	Cow's milk (1)	%DV^*^	Non‐transgenic soy drink (2)	%DV^*^	Transgenic soy drink (3)	%DV^*^
Energy value	529 (kJ)	6	399 (kJ)	5	290 (kJ)	3
Carbohydrates	10 (g)	3	12 (g)	4	2.3 (g)	1
Proteins	7.0 (g)	9	5.4 (g)	7	6.4 (g)	9
Total fats	6.4 (g)	12	2.9 (g)	5	3.6 (g)	7
Saturated fats	4.0	18	1.7 (g)	8	0.7 (g)	3
Sodium	120 (mg)	5	95 (mg)	4	178 (mg)	7
Calcium	240 (mg)	24	240 (mg)	24	264 (mg)	26
Vitamin A	—	—	—	—	162 (mcg)	27
Vitamin D	—	—	—	—	2.8 (mcg)	56
Vitamin E	—	—	—	—	2.7 (mg)	27
Vitamin B6	—	—	—	—	0.22 (mg)	17
Folic acid	—	—	—	—	1.2 (mcg)	50
Zinc	—	—	—	—	1.1(mcg)	16
Vitamin B12	—	—	—	—	1.2 (mcg)	50

* %(DV) indicates daily values are based on a 2000 kcal.

### 2.3 Bone Biometric Analysis

The bone anatomical dimensions were measured using a universal analog caliper. The following measurements were obtained: (i) femur length, (ii) proximal femur width, (iii) distal femur width, and (iv) femur diaphysis width.

### 2.4 Chemical Elements Assay by Energy Dispersive X‐Ray Spectroscopy

The mineral content of the femoral diaphysis and epiphysis was analyzed by energy‐dispersive x‐ray spectroscopy (EDS) using a scanning electron microscope with an x‐ray detection system. The bone fragments were immersed in fresh histological fixative (2.5% glutaraldehyde, 0.2% picric acid, 3% sucrose, and 5 mM CaCl_2_ prepared in 0.1 M sodium cacodylate buffer, pH 7.2), dehydrated in ethanol, and subjected to critical point drying (CPD030; Bal‐tec, Witten, North Rhine‐Westphalia, Germany), followed by carbon coating (Quorum Q150 T, Laughton, East Sussex, UK). The analysis was performed at ×500 magnification, measuring the proportions of calcium (Ca), phosphorus (P), manganese (Mn), copper (Cu), zinc (Zn), selenium (Se), magnesium (Mg), and sulfur (S), which were expressed as mean values for all analyzed areas.

### 2.5 Bone Histomorphometric Analysis

The distal epiphyses of the right femurs were decalcified by immersion in a decalcifying solution containing formic acid (20% diluted in distilled water) for 30 days until complete decalcification of the material was achieved [22]. The anteroposterior thickness of the epiphysis was measured and the medial portion was sectioned and embedded in paraffin. Four‐micrometer‐thick sections were stained with hematoxylin and eosin (H&E) for histomorphometric analysis and with Sirius Red for collagen fibers (I and III) analysis. All slides were examined using ×20 and ×40 objective lenses, and digital images were captured using a brightfield photomicroscope. For bone diameter (BD), medullary canal diameter (DM), and cortical thickness (CT), five measurements were made in various directions using the image analysis software Image‐Pro Plus 4.5 [23]. As reported by Sequetto [24], bone area (B.Ar), trabecular width (Tb.Wi), and trabecular separation (Tb.Sp) were analyzed in 10 regions of interest (ROI) per animal at ×200 magnification. All measurements were performed using ImageJ software with the BoneJ plugin [25, 26], specifically applying the sphere‐fitting method for Tb.Wi and Tb.Sp quantification.

### 2.6 Collagen Analysis

Collagen fibers were analyzed using the Sirius Red staining method and polarized light microscopy, following previously reported methodology [27, 28]. The volume density of collagen fibers was estimated based on their birefringence properties (type I: red‐yellow; type III: green), as described by Sarandy [29].

### 2.7 Scanning Electron Microscopy

The bone fragments were immersed in fresh histological fixative (in 2.5% glutaraldehyde, 0.2% picric acid, 3% sucrose, and 5 mM CaCl_2_ prepared in 0.1 M sodium cacodylate buffer, pH 7.2), subjected to critical point drying in ethanol and gold coating, following previously reported methodology [30]. The analysis was performed on fragments of the proximal, mid, and distal femoral diaphysis. Five random fields (×3000 magnification) per segment were evaluated. Cortical bone porosity (N/mm^2^ and %) were quantified as described by Sarandy [29].

### 2.8 Bone Biomechanical Assay

Biomechanical analysis was performed on the left femur using a three‐point bending test on the EMIC Universal Testing Machine (Bioengineering Laboratory, FMRP/USP, Ribeirão Preto, Brazil). Femurs were stored at −20°C, then tested after thawing. A 500 N load cell was used with progression of 1 mm/min until mechanical failure. Tesc software generated load versus displacement graphs to determine maximum force, relative stiffness, and displacement.

### 2.9 Immunofluorescence Analysis

Immunofluorescence analysis was performed on bone epiphysis fragments fixed in 4% paraformaldehyde for 24 h. After fixation, samples were washed, incubated with anti‐osteocalcin primary antibody (1:100 dilution) and FITC‐conjugated secondary antibody (1:1000 dilution), and counterstained with 4',6‐diamidino‐2‐phenylindole (DAPI). The sections (3 µm thickness) were analyzed using an EVOS M5000 microscope (×20 objective). Osteocalcin‐positive cells were quantified using Image Pro‐plus software (version 7.0.1).

### 2.10 Statistical Analysis

Data were expressed as mean and standard deviation of the mean (mean ± S.D.). Data normality was investigated using the Kolmogorov–Smirnov test. Parametric data were analyzed by One‐Way ANOVA and Tukey's post hoc test, while non‐parametric data were evaluated by the Kruskal–Wallis method. Results with *p* ≤ 0.05 were considered statistically significant.

## 3 Results

### 3.1 Animal's Weight

As shown in Table 2, initial and final body mass was similar in all groups (*p* > 0.05). All animals gained weight, but no significant differences were observed between G1, G2, and G4 (*p* > 0.05). On the other hand, G3 presented the lowest body mass gain compared to G1 and G4 animals (*p* < 0.05) (Table 2).

**TABLE 2:** Variation of weight in mice untreated and treated with cow's milk, non‐transgenic, and transgenic soy drink.

Groups	Initial weight (g)	Final weight (g)	Weight gain (g)
G1	34.51 ± 2.98	36.17 ± 2.64	1.66 ± 0.34^a^
G2	31.61 ± 2.72	32.70 ± 2.59	1.09 ± 0.13^a.b^
G3	30.92 ± 3.08	30.95 ± 2.70	0.03 ± 0.38^b^
G4	33.70 ± 2.22	35.74 ± 1.97	2.04 ± 0.25^a^

*Note*: (A) G1: distilled water; G2: non‐transgenic soy drink; G3: transgenic soy drink; G4: whole cow's milk. Data are expressed as mean ± standard deviation. (B) a,b Different letters in the rows indicate statistical differences (*p* < 0.05) between the groups. Groups with the same letter in the columns do not show statistical differences (*p* > 0.05).

### 3.2 Biometric Characteristics

**FIGURE 1:**
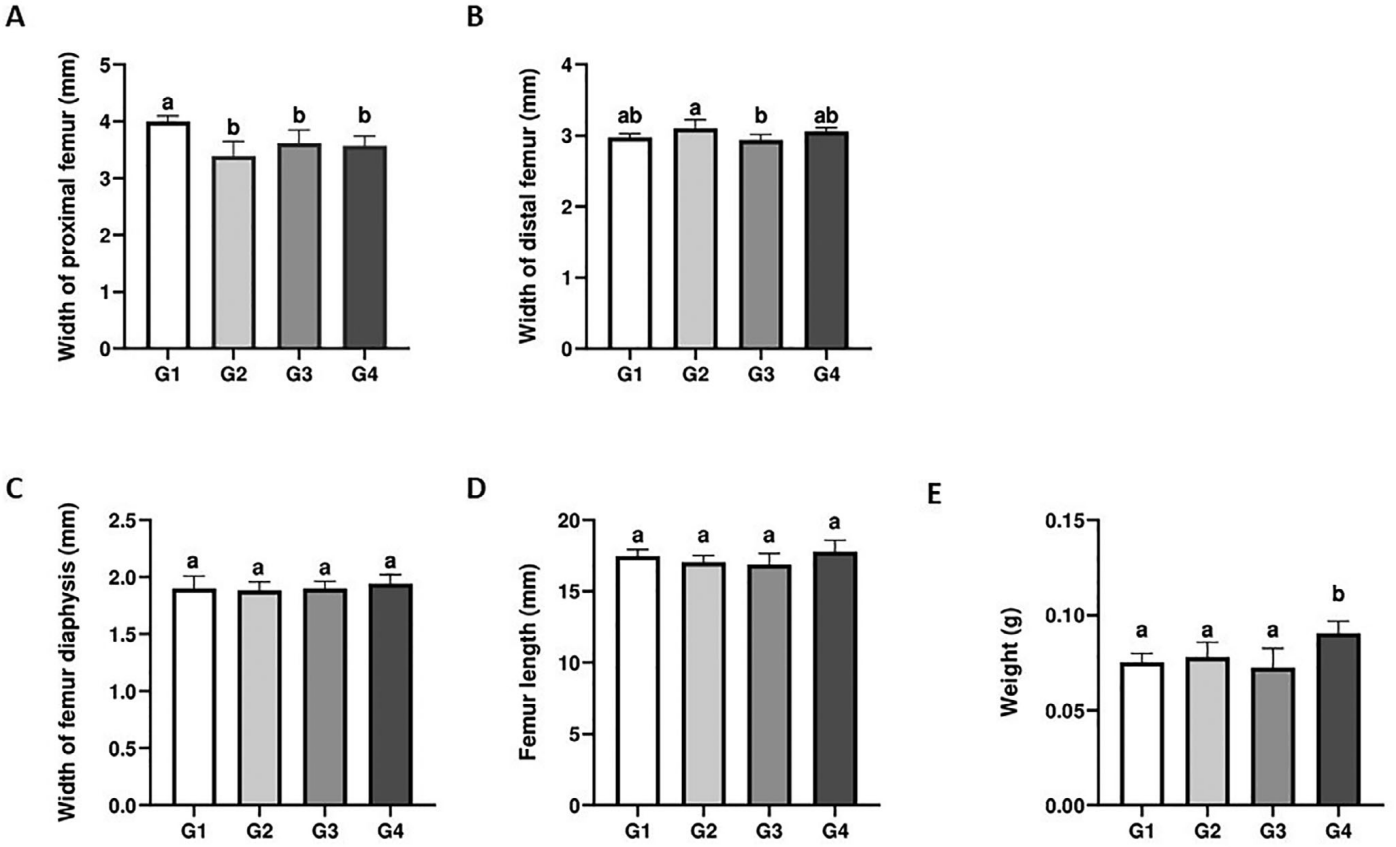
Anatomical dimensions of the femur in untreated mice and those treated with cow's milk, non‐transgenic, or transgenic soy beverages. (A) proximal femur width, (B) distal femur width, (C) diaphysis width, (D) femur length, and (E) femur weight. Different letters indicate significant differences between groups (*p* < 0.05).

The anatomical parameters showed that proximal femur width was reduced in G2, G3, and G4 compared to control animals (*p* < 0.05) (Figure 1A). Animals treated only non‐transgenic soy drink (G2) presented higher distal femur width compared to the group receiving only transgenic soy drink (G3) (*p* < 0.05). No significant differences between G2 and G3 were observed when compared to G1 and G4 (Figure 1B). Femur diaphysis width and femur length were similar in all experimental groups (*p* > 0.05) (Figure 1C–E). In addition, femur weight was increased in G4 compared to the other groups (*p* < 0.05) (Figure 1E).

### 3.3 Chemical Elements Distribution

Quantitative analysis of minerals in the femurs revealed that animals treated with cow's milk (G4) presented a higher magnesium content compared to control animals (G1) (*p* < 0.05). Phosphorus levels were similar comparing G1 with the other groups (*p* > 0.05). However, increased phosphorus levels were identified G4 compared to G3 animals (*p* < 0.05). Calcium levels were increased in all treated groups compared to control animals (G1) (*p* < 0.05). Similar calcium content was observed in G2, G3, and G4 (*p* > 0.05). Manganese, copper, zinc, selenium, and sulfur were similarly distributed in all groups (*p* > 0.05) (Table 3).

**TABLE 3:** Mineral content in the femur of mice treated with distilled water (G1); non‐transgenic soy drink (G2), transgenic soy drink (G3), and cow's milk (G4).

Element (%)	G1/	G2	G3	G4
Mg	1.48 ± 0.41^a^
S	2.14 ± 1.42	1.98 ± 1.41	2.80 ± 1.17	2.02 ± 0.63
P	33.59 ± 7.3^ab^	35.69 ± 4.19^ab^	31.78 ± 0.90^a^	37.94 ± 3.55^b^
Ca	33.15 ± 18.9^a^	57.13 ± 3.55^b^	60.21 ± 2.32^b^	61.31 ± 2.35^b^
Mn	0.40 ± 0.19	0.28 ± 0.17	0.43 ± 0.24	0.34 ± 0.22
Cu	0.42 ± 0.21	0.34 ± 0.18	0.51 ± 0.22	0.22 ± 0.04
Zn	0.53 ± 0.26	0.54 ± 0.20	0.49 ± 0.27	0.47 ± 0.17
Se	1.18 ± 0.74	1.17 ± 0.74	1.73 ± 1.23	1.02 ± 0.977

*Note*: (A)Mg: magnesium; S: sulfur; P: phosphorus; Ca: calcium; Mn: manganese; Cu: copper; Zn: zinc; Se: selenium. (B) Data are expressed as mean ± standard deviation. ^a,b^ Different letters in the rows indicate statistical differences (*p* < 0.05) between the groups. Groups with the same letter do not present statistical differences (*p* > 0.05).

### 3.4 Bone Microstructural Characteristics

**FIGURE 2:**
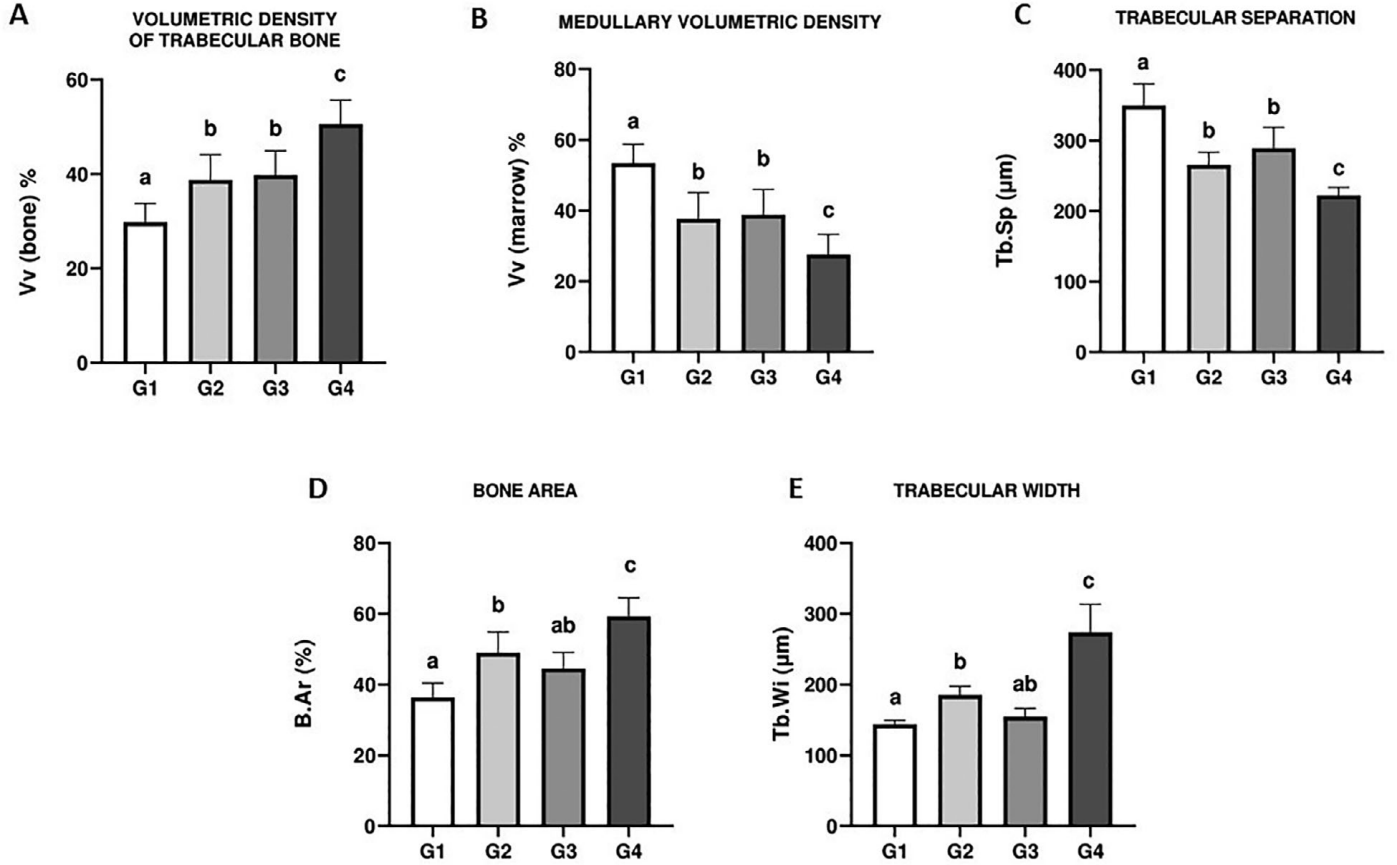
Trabecular bone microarchitecture in mice untreated or treated with cow's milk, non‐transgenic, or transgenic soy beverages. (A) Volumetric density of trabecular bone (Vv bone), (B) medullary volumetric density (Vv medulla), (C) trabecular separation (Tb.Sp), (D) bone area (B.Ar), and (E) trabecular width (Tb.Wi). Different letters indicate significant differences between groups (*p* < 0.05).

**FIGURE 3:**
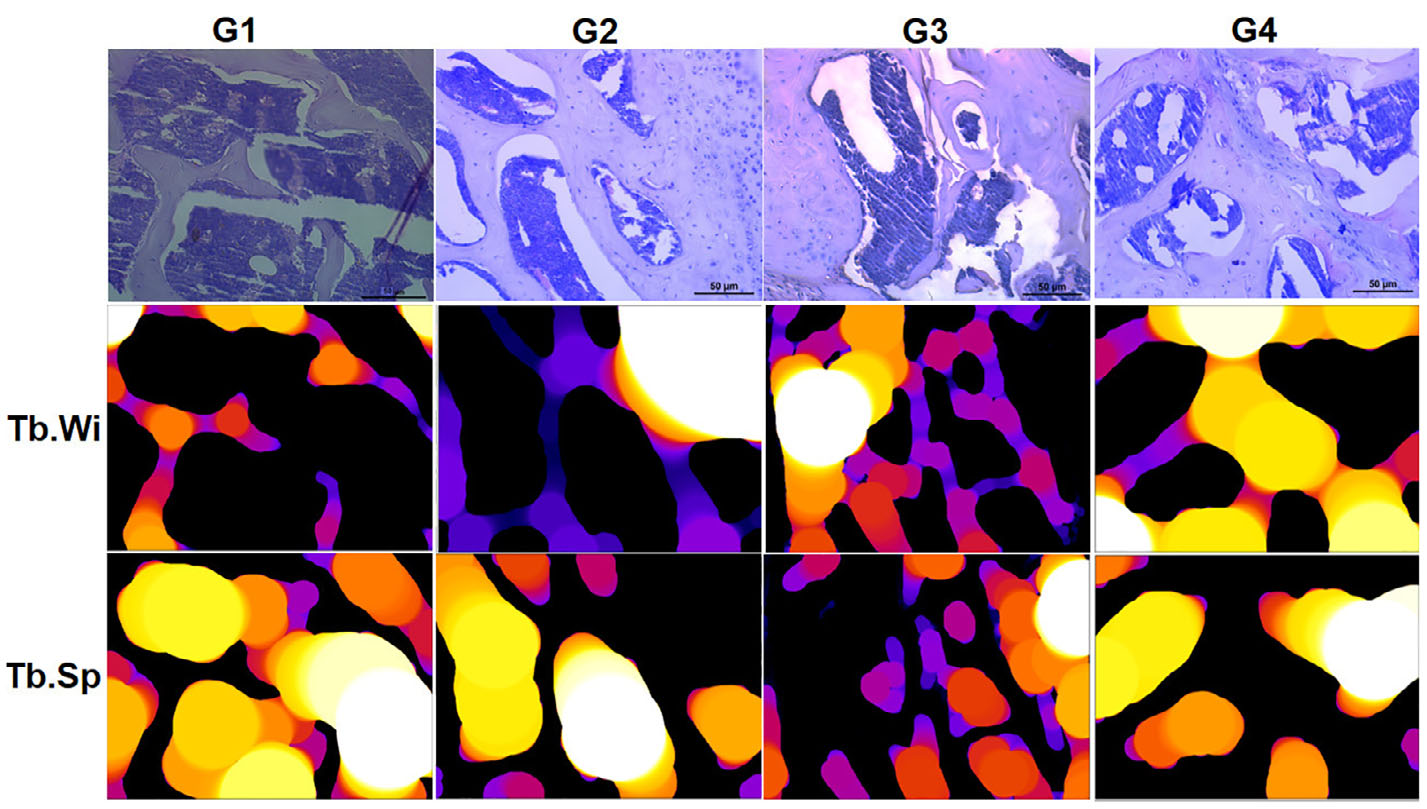
Sphere‐fitting method for calculating trabecular bone width (Tb.Wi) and trabecular separation (Tb.Sp) using ImageJ. Top row: hematoxylin–eosin‐stained sections observed under bright‐field microscopy. Different colors indicate spheres positioned within and between trabeculae.

Femur microstructure is shown in Figures 2 and 3. Increased trabecular volumetric density was observed in all treated groups (G2–G4) compared to control animals (G1) (*p* < 0.05). This parameter was similar in G2 and G3 (*p* > 0.05), but was higher in G4 compared to the other groups (*p* < 0.05) (Figure 2A). G2, G3, and G4 animals exhibited reduced medullary volumetric density and trabecular separation compared to control mice (G1) (*p* < 0.05). These parameters were even more reduced in G4 compared to the other groups (*p* < 0.05). Bone area and trabecular width were similar in G1 and G3 (*p* > 0.05), but increased in G4 compared to the other groups (*p* < 0.05) and in G2 compared to control animals (G1) (*p* < 0.05).

Figure 3 illustrates the application of the sphere‐fitting algorithm in the BoneJ plugin (ImageJ), used to calculate Tb.Wi and Tb.Sp. The colored spheres represent the measurement of trabecular thickness and separation, offering a visual demonstration of how these parameters were obtained from the microstructural images.

**FIGURE 4:**
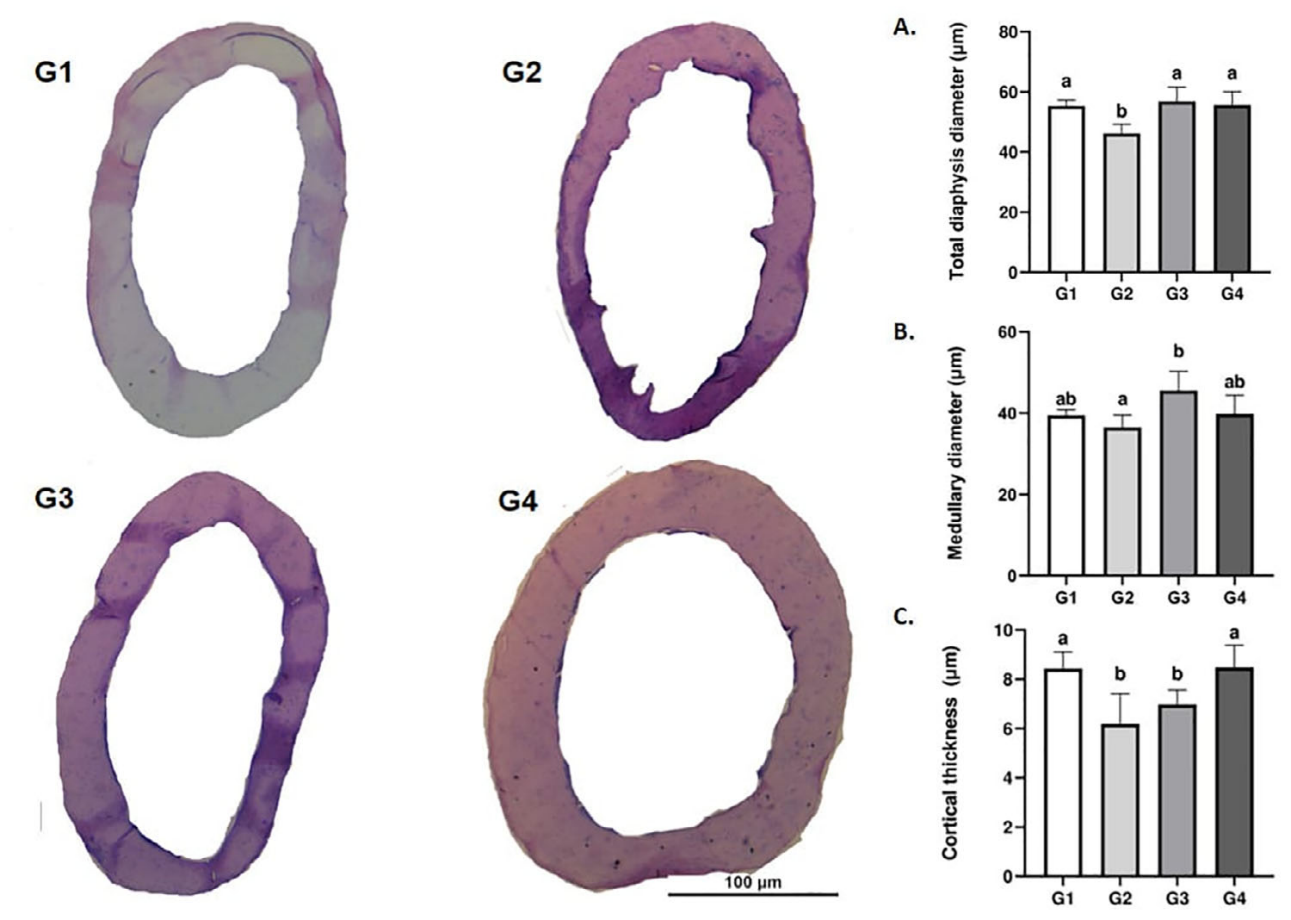
Representative microscopic images of the femoral diaphysis (hematoxylin–eosin staining, bright‐field microscopy) and diaphyseal morphometric parameters. (A) Diaphysis diameter, (B) medullary diameter, and (C) cortical thickness. Different letters indicate significant differences between groups (*p* < 0.05).

The microstructural parameters of the femoral diaphysis are presented in Figure 4. Diaphysis diameter was reduced in G2 compared to the other groups (*p* < 0.05). The medullary diameter in all groups was similar to the control (G1). This parameter was higher in G3 compared to G2 (*p* < 0.05). Cortical bone thickness was reduced in G2 and G3 (*p* < 0.05) compared to G1 and G4, which exhibited similar values (*p* > 0.05).

### 3.5 Collagen Analysis

**FIGURE 5:**
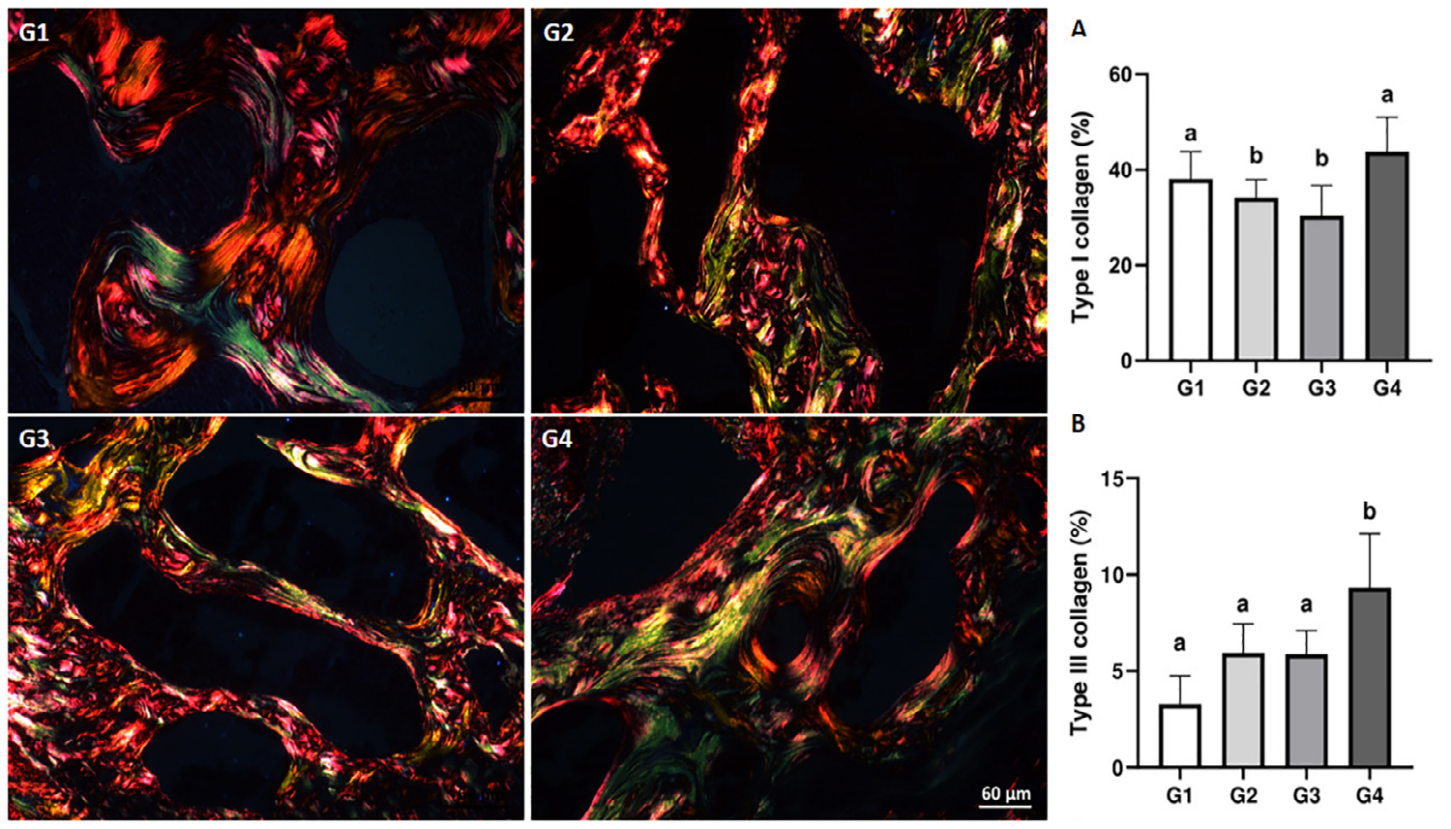
Representative microscopic images and quantification of type I (A) and type III (B) collagen in the femoral epiphysis (Sirius Red staining, polarized light microscopy). Type I fibers appear in yellow/orange/red, type III fibers in green, and the medullary space in black. Different letters indicate significant differences between groups (*p* < 0.05).

As shown in Figure 5, Type I collagen content was reduced in G2 and G3 compared to G1 and G4 (*p* < 0.05). Conversely, this parameter was similar in G1 and G4 (*p* > 0.05). In addition, increased type III collagen (Figure 5B) distribution was observed in G4 compared to the other groups (*p* < 0.05). Type III collagen content was also similar in G1, G2, and G3 (*p* > 0.05).

### 3.6 Bone Porosity

**FIGURE 6:**
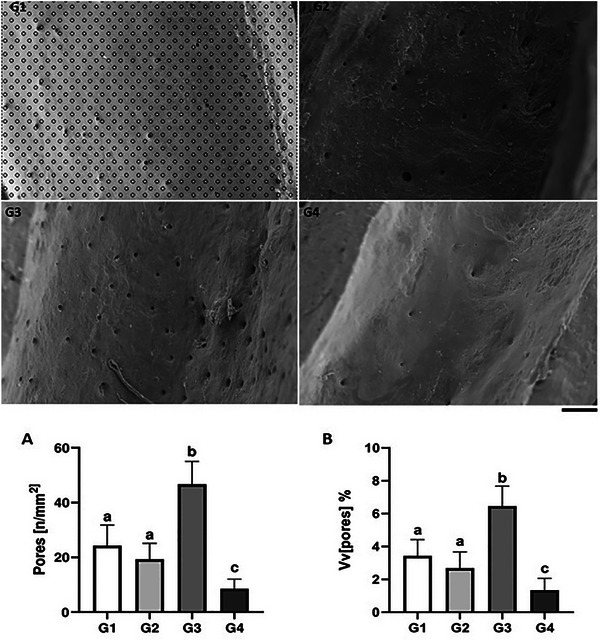
Scanning electron microscopy showing the method used to determine pores in the femoral diaphysis (scale bar = 200 µm). (A) Number of pores per histological area and (B) volume density of pores (Vv). Different letters indicate significant differences between groups (*p* < 0.05).

Scanning electron microscopy images revealed bone porosity of the femoral diaphysis in the different experimental groups (Figure 6). The number of pores per histological area and the proportion of bone tissue occupied by pores was reduced in G4 compared to the other groups (*p* < 0.05). Conversely, increased porosity was observed in G3 compared to G1, G2, and G4 (*p* < 0.05). These parameters were similar in G1 and G2 (*p* > 0.05).

### 3.7 Bone Biomechanical Function

**FIGURE 7:**
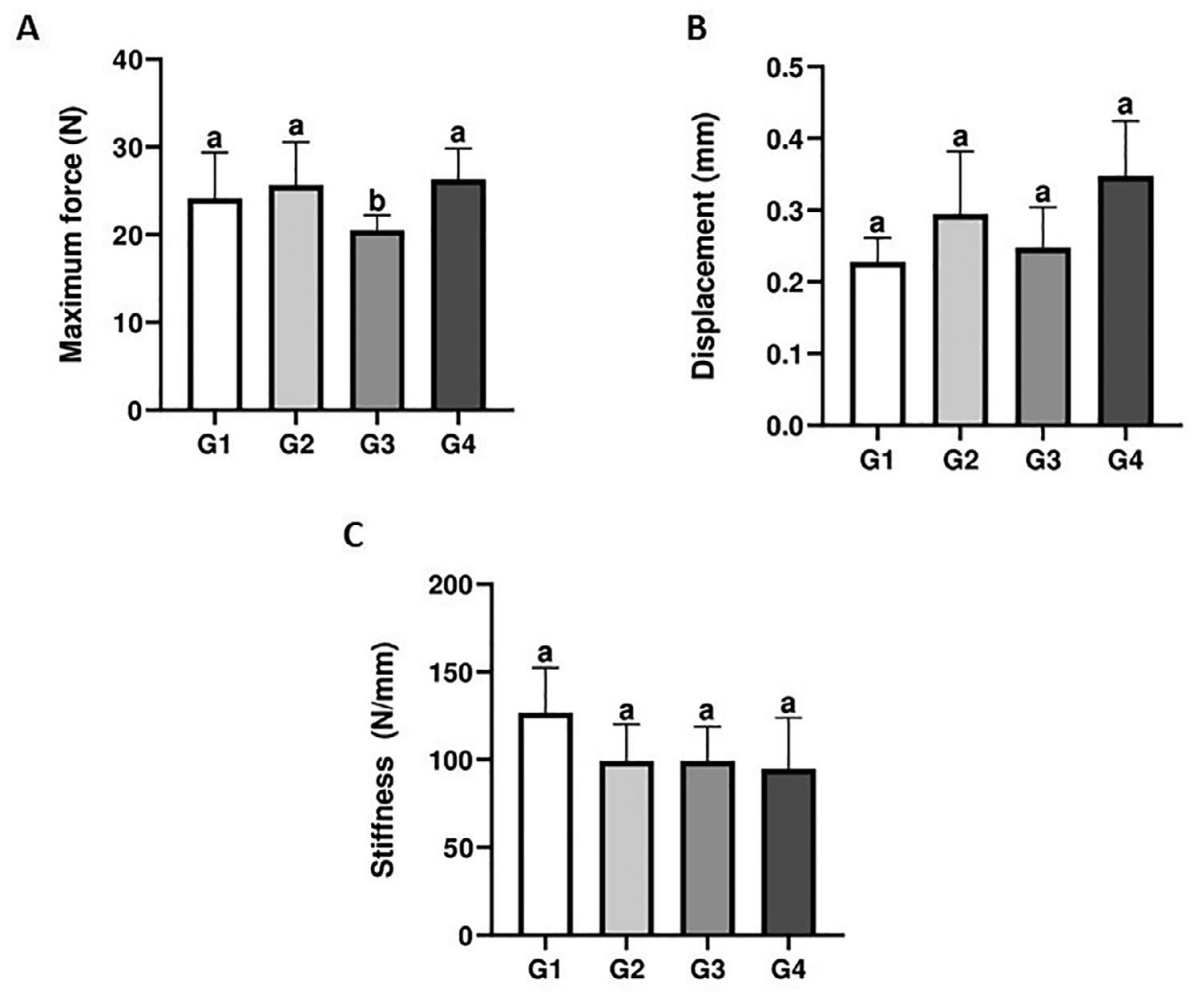
Mechanical resistance (A), displacement (B), and stiffness (C) of the femur. Different letters indicate significant differences between groups (*p* < 0.05).

As indicated in Figure 7, the ability to withstand maximum load was lower in animals that received the transgenic soy beverage (G3) compared to the other groups (*p* < 0.05). Maximum displacement to fracture and bone stiffness were similar in all groups (*p* > 0.05).

### 3.8 Bone Expression of Osteocalcin

**FIGURE 8:**
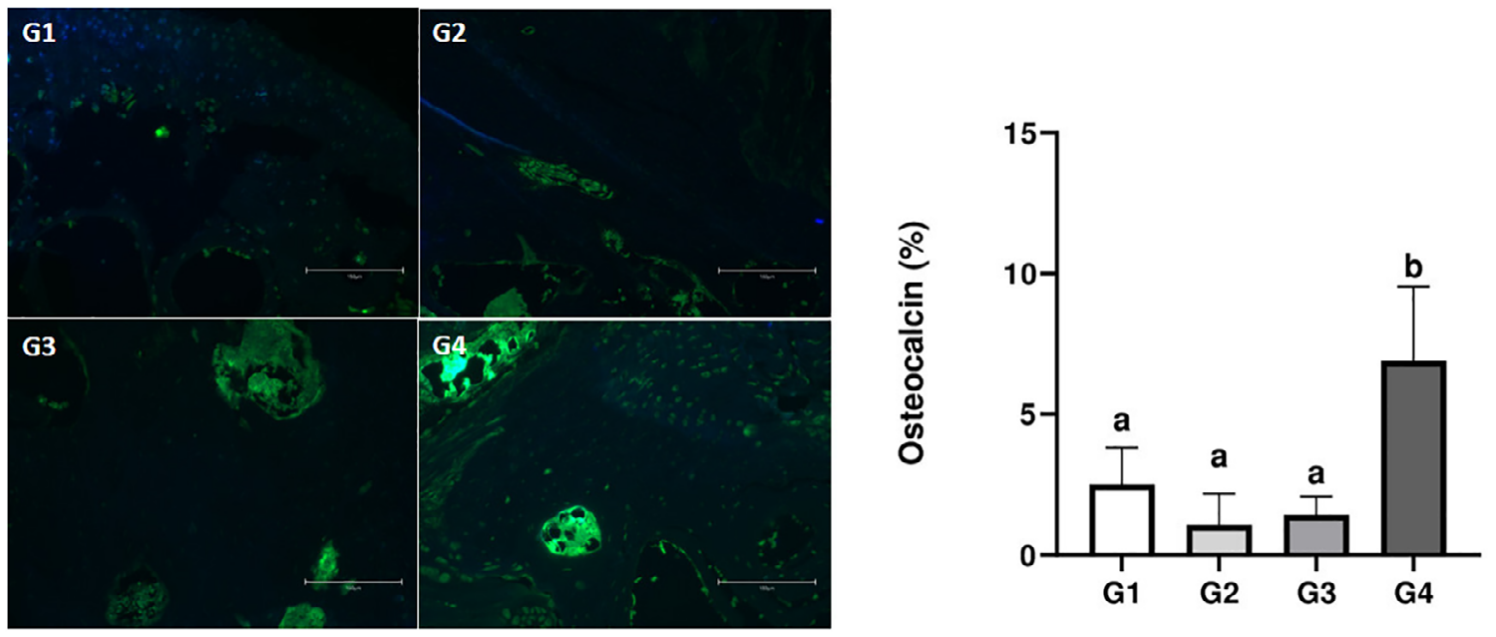
Osteocalcin immunostaining in the femoral epiphysis (fluorescence microscopy, left) and relative levels of osteocalcin (right). Different letters indicate significant differences between groups (*p* < 0.05).

The immunostaining of osteocalcin protein is shown in Figure 8. Immunohistochemical analysis showed greater positivity for osteocalcin in the group treated with cow's milk (G4) compared to the other groups (*p* < 0.05). Immunostaining pattern was similar in G1, G2, and G3 (*p* > 0.05).

## 4 Discussion

The nutritional compounds present in animal‐ and plant‐based foods and their health effects have been widely studied [31, 32, 33, 34, 35]. Soy‐based beverages are considered alternatives to animal‐derived products, but their health effects are still questionable [18, 32, 34]. Some studies suggest that soy influences body weight through isoflavones, which affect adipogenesis and lipid metabolism [36, 37]. Our study found that transgenic soy‐based beverages reduced animal weight compared to control and cow's milk groups, while non‐transgenic soy had no significant effect. This finding may be associated with heat‐stable antinutrients such as phytoestrogens, glucinins, and phytic acid [38, 39] or toxins from genetically modified plants [40]. However, further studies are needed to confirm or refute this hypothesis.

Our investigation further highlighted that these beverages affect not only body weight but also bone microarchitecture. The groups receiving both transgenic and non‐transgenic soy beverages showed beneficial effects on bone health, improving all trabecular microarchitecture parameters. These effects are likely attributable to the isoflavones present in soy, particularly genistein and daidzein, which are well‐documented for their estrogen‐like activity and bone‐protective properties. Isoflavones bind to estrogen receptors, especially ER‐β, which is more abundantly expressed in trabecular bone, possibly explaining the selective improvement in trabecular, but not cortical, bone parameters [41]. Several studies have shown that genistein and daidzein can increase osteoblast activity and reduce osteoclast differentiation, thus promoting bone formation and reducing bone resorption. Although we did not perform a detailed compositional analysis of the soy beverages, this is a recognized limitation. However, the observed beneficial outcomes are consistent with the established literature on soy isoflavones and bone metabolism, suggesting that these compounds likely contributed to the effects observed in both groups receiving the soy beverages. Bone remodeling is also modulated by oxidative stress and inflammation, as ROS and pro‐inflammatory cytokines stimulate osteoclastogenesis and impair osteoblast activity [42]. Isoflavones and milk‐derived peptides have reported antioxidant and anti‐inflammatory properties, which may partly explain their bone‐protective effects.

Additionally, cow's milk has demonstrated beneficial effects on trabecular microarchitecture, improving parameters such as trabecular volumetric density, bone area, width, and separation. Studies suggest that specific milk proteins and peptides play a role in bone remodeling by regulating cellular markers such as osteocalcin and pathways involved in osteoclast and osteoblast metabolism [3]. Consistent with these findings, a study using young mice showed that supplementation with isolated milk proteins significantly increased femoral trabecular volume compared to untreated controls [43]. Furthermore, Dongen [44] reported that higher milk intake, particularly yogurt and cheese, increased trabecular volumetric density in men.

The integrity of cortical bone is essential for bone strength, and changes in its thickness or porosity can increase fracture susceptibility. Its growth results from the coordinated activity of osteoblasts and osteoclasts on the periosteal (outer) and endosteal (inner) surfaces [45]. Periosteal apposition, mediated by osteoblasts, promotes the deposition of new bone matrix on the external surface, while endosteal resorption, carried out by osteoclasts, degrades the bone matrix on the internal bone surface, regulating the cortical thickness and medullary cavity diameter [46]. Under physiological conditions, these processes remain balanced, ensuring skeletal integrity [47]. However, increased endosteal resorption combined with reduced periosteal apposition can lead to cortical thinning and medullary cavity expansion [48]. In the present study, G3 animals exhibited reduced cortical thickness and increased medullary cavity diameter, suggesting an imbalance in bone remodeling that may compromise bone strength. In contrast, animals that received cow's milk presented diaphyseal diameter, cortical thickness, and medullary area similar to control animals, indicating preservation of the balance between bone resorption and apposition. Likewise, G2 animals maintained a medullary cavity diameter comparable to control animals, suggesting that bone remodeling was not affected. Considering that increased nutritional demand for bone formation can lead to medullary cavity expansion [49], we hypothesize that transgenic soy‐based beverages may have increased the metabolic demand on bone tissue, potentially contributing to the structural alterations observed in the femur. However, given the lack of detailed compositional analysis, these findings should be interpreted cautiously and regarded as hypothesis‐generating. While genetically modified foods may offer benefits, they may also contain antinutritional factors or bioactive compounds with potential health effects [38].

Cortical bone structure is crucial for mechanical strength and impact absorption capacity, and porosity in this region can be an indicator of bone loss [50]. Increased cortical porosity is commonly attributed to an imbalance in bone remodeling, where resorption exceeds formation. This increase in cortical porosity is also associated with a decrease in bone stiffness and toughness [51]. In our study, we observed increased porosity in the compact bone of transgenic soy drink‐treated animals. Based on the histological analysis, we can infer that this group presented an imbalance in bone remodeling, leading to this increased porosity. Furthermore, bone porosity was reduced in cow's milk‐treated animals, indicating an acceleration in bone microstructure restoration, which is consistent with the results observed in trabecular bone, where trabecular volumetric density, bone area, and trabecular width also increased compared to the control group.

Collagen accounts for 90% of bone matrix proteins, playing a fundamental role in the properties and functions of this tissue [52]. Type I collagen is the main organic bone component, contributing to mineralization [5] and providing greater tensile strength [53]. Type III collagen is present especially in tendon and ligament bone insertions [54]. Although ignored in some research, bone proteins are fundamental for bone mineralization and growth, as they form the morphofunctional basis of these processes [22]. Here, both transgenic and non‐transgenic soy beverages were detrimental, decreasing type I collagen fibers, indicating a negative osteogenic effect, since the main function of type I collagen in the bone extracellular matrix is structural, and the assembly and biochemical characteristics of these fibers interfere with bone mechanical properties [55]. On the other hand, animals that received cow's milk maintained the type I collagen fibers pattern similar to the control group, as well as restored type III collagen fibers, indicating a positive role in osteoblastogenesis regulation, trabecular bone formation, and maintenance [56]. In addition, studies show that type III collagen plays an important role in bone repair by preserving the osteogenic potential of stem cells, stimulating angiogenesis [57].

Calcium and magnesium are essential minerals for bone health [58]. Calcium, a key component of mammalian bone tissue, regulates critical extracellular and intracellular processes involved in bone development [59]. While cow's milk is a natural calcium source, soy‐based beverages require fortification, commonly with calcium phosphate [34]. According to product labeling, the soy beverages used in this study (transgenic and non‐transgenic) were fortified with tricalcium phosphate, a common additive in plant‐based milk alternatives. Although these fortified beverages can reach total calcium concentrations similar to those found in cow's milk, tricalcium phosphate bioavailability is generally lower, due in part to the absence of casein phosphopeptides (CPPS), which increase calcium solubility and intestinal absorption. Furthermore, the phytates naturally present in soy can bind to calcium, forming insoluble complexes that further reduce its bioavailability [60, 61]. In our study, G2, G3, and G4 animals showed increased calcium concentrations compared to control animals, highlighting the importance of evaluating the nutritional composition of different calcium sources. Notably, only animals fed cow's milk exhibited a significant increase in magnesium levels, reinforcing evidence that dairy products are rich sources of this mineral, which plays a crucial role in bone regeneration and angiogenesis. Reducing dietary magnesium intake may impair bone metabolism. Accordingly, dairy foods such as milk contribute to the intake of essential nutrients in the diet such as magnesium, while demonstrating that fortified soy beverages are not a significant source of many dairy nutrients, including this mineral [62, 63]. A systematic review and meta‐analysis evaluating magnesium impact on bone health in older adults, higher magnesium intake was associated with increased bone mineral density in the femoral neck [64]. Therefore, we believe that the increase in magnesium levels had a beneficial impact on the bone health of animals receiving cow´s milk, especially on trabecular microarchitecture. Inorganic elements, such as Zn, Cu, P, Se, and S, are also fundamental for bone metabolism, influencing osteogenesis and bone resorption [28, 65]. Their similar concentrations between groups suggest preserved metabolic activity in bone tissue, regardless of dietary intervention [58].

Bone strength is determined as the maximum capacity to withstand forces before fracture or structural failure occurs [66, 67]. Several factors contribute to bone strength and are considered determinants of bone quality, including overall bone structure, which is determined by tissue mass and distribution. Bone impairment can be caused by decreased bone mass, changes in bone microarchitecture or geometry, including increased cortical porosity and tissue composition, such as the proportion of type I collagen and non‐collagenous proteins [68, 69]. In addition, factors such as cortical bone thickness and medullary diameter play an important role in determining bone strength [51]. Considering the results of the previous analyses, we believed that the transgenic soy beverage would compromise bone strength, as the animals receiving this beverage had greater cortical porosity, lower amounts of type I collagen, and also a larger diameter of the cortical medullary region, indicating a possible imbalance between resorption and apposition. Similar to our findings, studies on human bones with denatured type I collagen showed that they lost strength compared to undenatured bones, indicating that these changes may lead to an increased fracture risk [6]. After performing mechanical tests, the hypothesis that genetically modified soy beverage would compromise bone strength was confirmed by our results, indicating that this beverage may have a negative effect on tissue quality and strength. As a limitation, only the three‐point bending test was performed. Future studies should consider additional biomechanical approaches, such as torsion, compression, or nanoindentation, to provide a more comprehensive characterization.

Osteocalcin is the major non‐collagenous protein (NCP) produced by osteoblasts and is a specific marker of bone formation and resorption [70]. This protein modulates bone mass and is involved in the organization of the extracellular matrix and coordination of cell–matrix interactions, regulating bone structure and morphology [71]. Some studies suggest that increased osteocalcin levels stimulate bone formation by osteoblasts and, when combined with calcium ions, exert a positive effect on bone deposition and growth [72, 73]. This is consistent with our findings, as animals receiving cow's milk showed increased osteocalcin levels and a corresponding increase in calcium levels compared to the control group. More intense immunostaining suggests better bone repair [74], a characteristic observed in the group receiving cow's milk. When osteocalcin levels increase, osteoblast activity and bone remodeling accelerate. Mechanistically, the increase in osteocalcin in the femur of cow's milk‐treated animals suggests that milk may increase the number of osteoblasts or enhance the activity of existing cells. Cow's milk may exert anabolic effects on bone by providing casein phosphopeptides (CPPs) and milk basic protein (MBP). CPPs stimulate calcium absorption and directly stimulate osteoblast differentiation through the MAPK and Wnt/β‐catenin signaling pathways, while MBP has been shown to increase osteocalcin expression and bone mineral density in animal models and humans [3, 75]. These components, together with the high bioavailability of calcium and phosphorus in milk, can synergistically stimulate osteoblast proliferation and differentiation, thus supporting osteocalcin production, bone matrix mineralization, and bone remodeling. Such effects may also contribute to osteoclast depletion (responsible for bone resorption) and promote an increase in bone formation [73], which explains the increased trabecular volumetric density, Tb.Wi, and B.Ar, as well as the lower medullary volumetric density and Tb.Sp in cow's milk‐treated animals. Consistent with our results, a study in healthy women showed that milk basic protein (MBP) supplementation increased bone mineral density and improved bone metabolism. In this study, osteocalcin concentration was higher in the MBP group than in the control group after 6 months of intervention, indicating an increase in bone formation rate [75]. This reinforces our findings that dietary modulation of the osteocalcin pathway plays a central role in bone metabolism regulation, justifying the conclusion that cow's milk exerts a superior regulatory effect on bone formation compared to soy‐based beverages. In addition to osteocalcin, which was the primary focus of our study, other molecular pathways are critically involved in skeletal homeostasis. Markers such as Runx2 and alkaline phosphatase (ALP) are central to osteoblast differentiation and mineralization, while the RANKL/OPG system and tartrate‐resistant acid phosphatase (TRAP) regulate osteoclast activity and bone resorption [76]. Future studies evaluating these markers could provide greater mechanistic depth and clarify how different dietary protein sources influence the balance between bone formation and resorption.

Although this study used BALB/c mice, a well‐established model for bone metabolism due to their physiological similarities with humans, our findings provide valuable insights into the potential effects of cow's milk and soy‐based beverages on bone health in humans. These results may help inform dietary recommendations, especially for populations at risk for bone diseases such as osteoporosis. From a translational perspective, our findings align with clinical evidence showing that dairy intake is associated with improved bone mineral density and reduced fracture risk in humans, particularly due to the bioavailability of calcium, magnesium, and bioactive peptides [43, 75]. In contrast, the effects of soy on human bone health remain controversial, with some studies reporting benefits of isoflavones on trabecular bone in postmenopausal women, while others show limited or no effects [74]. The present results reinforce these observations by demonstrating that milk exerts a stronger effect on osteocalcin expression and bone strength, whereas soy beverages, although beneficial for trabecular parameters, may have limitations related to collagen deposition and cortical structure. Thus, our experimental model provides mechanistic support for clinical findings, underscoring the need for further studies directly comparing dairy and soy in human bone health. However, some limitations should be acknowledged, including the relatively small sample size, the exclusive use of male animals, which prevents the evaluation of possible sex‐dependent effects, the short intervention period, and the limited biochemical characterization of soy beverages. Future studies involving both sexes, longer interventions, and more diverse populations are needed to validate and expand our findings.

## 5 Concluding Remarks

Our findings provide a broader perspective on the role of dietary components in bone metabolism by comparing cow's milk, transgenic soybean, and non‐transgenic soybean. Beyond describing differences in bone microarchitecture and osteocalcin levels, our study underscores distinct mechanistic pathways: milk‐derived bioactive peptides appear to stimulate osteocalcin production and promote osteoblast activity, while soy isoflavones, particularly from non‐transgenic sources, likely act through estrogen receptor‐β to preserve trabecular bone integrity and regulate osteoclastogenesis. This mechanistic contrast highlights the novelty of our work, as few studies have directly compared the bone effects of genetically modified and unmodified soybeans against an animal protein reference. Collectively, these results suggest that dietary protein sources differentially shape bone remodeling via unique molecular targets, reinforcing the importance of understanding plant‐ and animal‐derived bioactives in bone health.

## Conflicts of Interest

The authors declare no conflicts of interest.

## Data Availability

All acquired data are systematically presented within the text.
